# Response to Impact of atrial high‐rate episodes and atrial cardiomyopathy on future stroke in patients with dual chamber permanent pacemakers

**DOI:** 10.1002/clc.23740

**Published:** 2021-10-09

**Authors:** Ju‐Yi Chen, Wei‐Da Lu

**Affiliations:** ^1^ Department of Internal Medicine, National Cheng Kung University Hospital, College of Medicine National Cheng Kung University Tainan Taiwan

To the Editor,

We would like to thank Dr. Miyo Nakano et al. for their kind interest in our article “The optimal cutoff of atrial high‐rate episodes for neurological events in patients with dual chamber permanent pacemakers”.^1^ As their previous articles,^2^, ^3^ they uncovered the important role of atrial cardiomyopathy (ACM) contributing to future neurological events (NE) in patients with permanent pacemakers. ACM^4^ has been proposed as a term to describe patients with abnormal atrial substrate and function, including atrial fibrosis, atrial electrical or mechanical dysfunction, and hypercoagulable state, which explains the possible mechanisms between atrial fibrillation (AF), even atrial high rate episodes (AHRE)^2^, ^3^ and systemic thromboembolism or embolic stroke of undetermined source.^5^ ACM could be evaluated by heart magnetic resonance imaging for detecting atrial fibrosis, P‐wave duration and maximum P‐wave area in a 12‐lead electrocardiography, and low pre‐implant left atrial emptying fraction detected by echocardiography.^6^ We have not mention ACM in our discussion because the temporal association^1^ and dissociation^6^ between AHRE and NE have been observed and we actually did not have evaluated ACM markers in our current study.^1^ Even though, we would like to thank for the suggestions from Dr. Miyo Nakano et al. We also hope that more and more evidences will be elucidated about the AHRE detected by pacemakers and NE in the future.
